# Observation of broad-band water waveguiding in shallow water: a revival

**DOI:** 10.1038/s41598-020-75335-8

**Published:** 2020-10-27

**Authors:** Fabián Sepúlveda-Soto, Diego Guzmán-Silva, Edgardo Rosas, Rodrigo A. Vicencio, Claudio Falcón

**Affiliations:** 1 Departamento de Física, Facultad de Ciencias Físicas y Matemáticas, Universidad de Chile, Casilla 487-3, Santiago, Chile; 2 ANID – Millenium Nucleus of Soft Smart Mechanical Metamaterials, Santiago, Chile; 3 ANID – Millennium Science Initiative Program – Millennium Institute for Research in Optics, Santiago, Chile

**Keywords:** Fluid dynamics, Optics and photonics, Mechanical engineering

## Abstract

We report on the observation and characterization of broad-band waveguiding of surface gravity waves in an open channel, in the shallow water limit. The waveguide is constructed by changing locally the depth of the fluid layer, which creates conditions for surface waves to propagate along the generated guide. We present experimental and numerical results of this shallow water waveguiding, which can be straightforwardly matched to the one-dimensional water wave equation of shallow water waves. Our work revitalizes water waveguiding research as a relevant and controllable experimental setup to study complex phenomena using waveguide geometries.

## Introduction

Energy localization and transport are major goals in physics due to their fundamental and applied impact^1^. In particular, waves allow the transport of energy/information from one region of space to another, by taking advantage of vibrational properties of the propagating media. This has been a key of success for the development of our modern societies where, for example, electrical waves propagate through copper cables transmitting information. Nowadays, different systems have consolidated as key technologies for the same tasks. Electromagnetic waves are broadly used for fast processing in open air as well as in solid materials^2,3^. Optical fibers, developed during the 70’s and consolidated during the 80’s and 90’s, form nowadays the most important communication network in our planet^4^. Almost all the internet global traffic is been transmitted through optical fibers across continents and oceans. Their operation is quite simple and it is nothing more than an optical solution to the wave equation, constrained to step-like refractive index potentials^4^. In fiber optics, light is trapped on a larger refractive index region. In that region, velocity is smaller and a gaussian-like profile is generated as a fundamental solution which propagates in a guided way along the fiber, presenting evanescent tails away from the fiber region. Depending on the specific conditions, optical fibers operate in single or multi-mode configurations, enhancing the possible ways of propagating energy/information by using orthogonal optical states, an important subject of research nowadays in order to increase the transmitted data volume by implementing different multiplexing techniques^5^. It is important to also mention that the inclusion of nonlinear interactions have allowed the observation of solitons in different optical configurations^6–8^, which can be viewed as a way to transmit information without requiring a waveguide structure due to self-trapping mechanisms.

**Figure 1:**
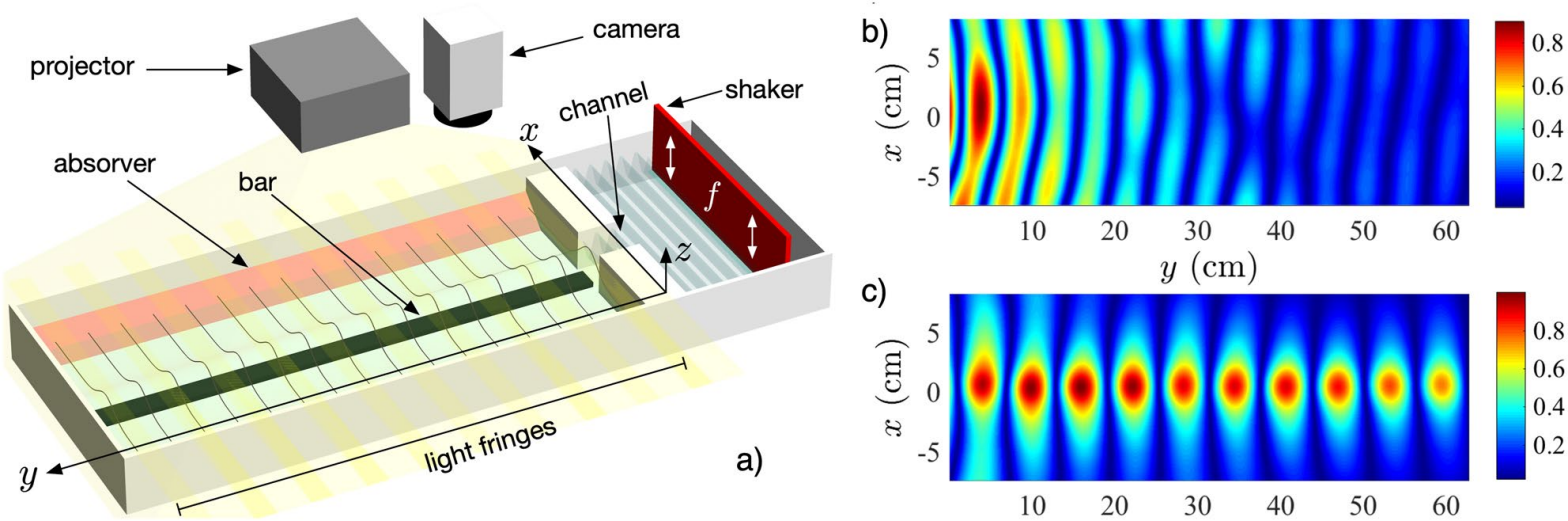
(**a**) Simplified experimental setup, including the measuring technique. (**b**) Wave amplitude in the open tank, without the waveguide. (**c**) Wave (guided) amplitude in the open tank, in the presence of a water waveguide with $h_g=1\,\hbox {cm}$. In both cases, $f_0= 3\,\hbox {Hz}$, $\delta \eta = 2\,\hbox {mm}$ and $a= 2.5\,\hbox {cm}$.

In the context of surface water waves, waveguiding has been observed spontaneously on planetary flows for Kelvin waves^9^ and edge waves along shores^10^. Historically, when looking for guiding solutions of surface waves, the first answer comes fast, and we simply focus our attention on the concept of *channelling* (what, in addition, includes the historical observation of a first nonlinear solitary wave^11^). Channelling has been quite an important contribution to our societies and, in fact, one of the keys of modern agronomy using controlled water supplies which become scarcer nowadays. In addition, channelling has been also an important step forward to avoid damages stemming from natural catastrophes such as floods or heavy seas. However, can we guide waves on open spaces like an ocean or a wide channel without channelling? This question started to be solved several years ago in different studies. For example, Arthur et al.^12^ suggested the analogy between ray optics and oceanographic waves in shallow and deep water limits, connecting optical waves concepts to water waves. In 1968, Longuett-Higgins^13^ theoretically suggested the possibility of trapping waves at oceanic depth discontinuities, which is related to optical evanescent waves developing due to total internal reflection. The same year, Buchwald^14^ described theoretically the waveguiding problem on an oceanic ridge, finding an infinite number of guided modes. This is probably the first theoretical prediction showing the possibility to guide a water wave without channeling, indicating a clear connection between optics and water wave phenomena. Interestingly, this ridge-induced localization could be a good explanation for several patterns observed in fluids when disordered ridge bottoms are present. In 1976, Stocks^15^ theoretically and experimentally described guided surface waves on the shallow-water limit, considering different bottom topographies and even a curved structure. This author nicely measured the fundamental guided mode by using a UV recorder and showed, for the first time, water wave trapping as a consequence of waveguiding and not due to channelling. No further attention on this kind of water waveguiding problems was reported and the topic was, surprisingly, forgotten. Almost forty years later, some theoretical and numerical work dealing with trapped wave modes on jet currents^16^ reprised the idea of water waveguiding, using a different approach due to the non-static nature of that problem.

Here, we explore water waveguiding in shallow water and describe a simple mechanism to control wave propagation based on waveguide concepts; i.e., to use the surface wave features in shallow fluid layers and observe waterguiding using a step-like potential. We follow a similar approach used more than 40 years ago^14,15^ to revitalize this research topic using modern measurement techniques, which allow us to calculate and track experimentally the broad-band of allowed propagating wave vectors. In order to guide a wave^17^, we require to define a spatial region experiencing a different velocity with respect to its surroundings. In the shallow water regime^18^, different depths imply different propagation velocities. This velocity contrast can generate guided solutions in the effective water-wave equation, allowing us to observe the propagation of guided water modes on a rather simple experimental setup. Our proposal uses an interesting way of controlling the propagation of water waves, which could be of great impact in basic and applied sciences. The ability to guide a wave is the elementary ingredient to study phenomena appearing, for example, in solid-state physics^19^ as well as in several physical contexts where different lattice geometries are an essential framework^20,21^, including recent studies on topological phenomena^22–26^, which includes water waves^27–29^ as well. Therefore, the idea of guiding and controlling a water wave opens new possibilities for physical research and, without a doubt, an emergent topic in basic experimental science that deserves to be revitalized to deepen its understanding.

## Experimental setup and results

Our surface wave experimental setup is depicted in Fig. 1a. A wave tank (200 cm long, 60 cm wide and 20 cm deep) is filled up to a height $h_0 = 2.5\,\hbox {cm}$ of destilled water. At its sides, absorvers (sponges) are placed to reduce wave reflections. At one end, 20 cm away from the wall, an electro-mechanical shaker, driven by a function generator via a power amplifier, generates monochromatic surface gravity waves. Waves with a frequency $f\in [2.0,5.0]\,\hbox {Hz}$ and an amplitude $\delta \eta \in [0.5,2.0]\,\hbox {mm}$ travel along a 10 cm plexiglass channel, creating a one-dimensional wave train. For a given *f* the wave trains display corresponding wavelengths $\lambda \in [6,16]\,\hbox {cm}$ in the channel. As the wave trains exit the channel and meet the open tank, diffraction occurs and a cylindrical front develops with a spatially decreasing amplitude due to radiation and viscous dissipation (see Fig. 1b). This observation significantly changes when a long bar is placed at the wave tank’s bottom, as sketched in Fig. 1a. Specifically, our bar has a height $h_g=1.5\,\hbox {cm}$, a width $a= 2.5\,\hbox {cm}$, and a length $l=100\,\hbox {cm}$. In this case (see Fig. 1c), wave trains propagate along the surface over the bar region, with a characteristic transversal profile. Waves do not change significantly for a distance much longer than the one showed for the open tank (see Fig. 1b). Thus, the observed water waves are well guided by a simple bathymetry reconfiguration without requiring channeling^11^.

**Figure 2:**
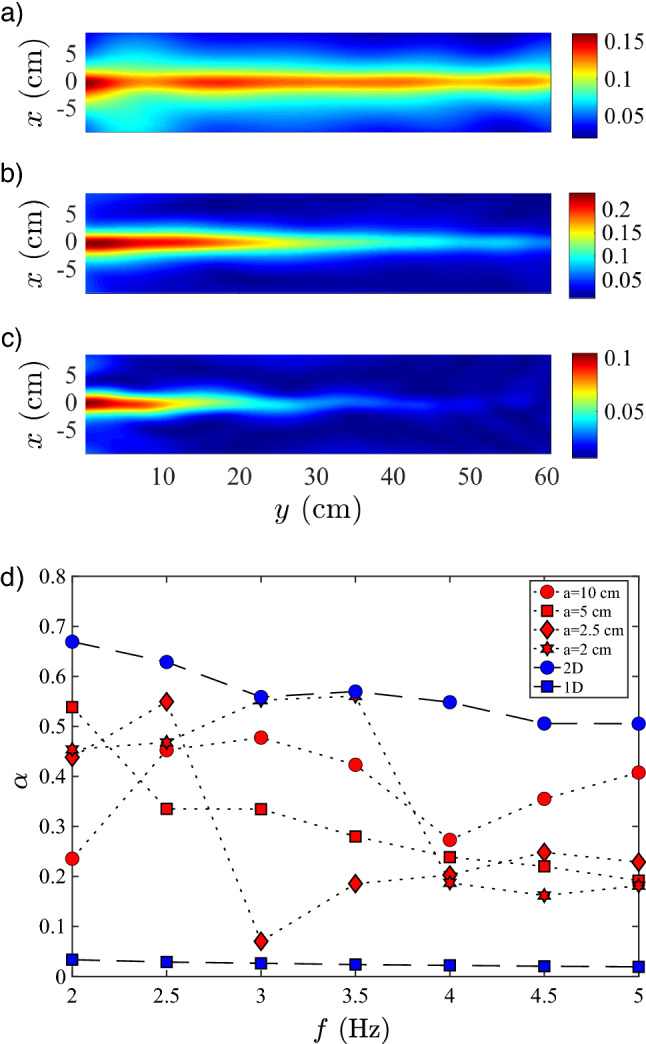
Wave envelope for (**a**) $f=2.5\,\hbox {Hz}$, (**b**) $f=3.5\,\hbox {Hz}$ and (**c**) $f=4.5\,\hbox {Hz}$. $a=2.5\,\hbox {cm}$ , $h_g=1.0\,\hbox {cm}$ and $\delta \eta =2\,\hbox {mm}$. (**d**) $\alpha$ values as a function of frequency $f_0\in$ [2.0,5.0] Hz for different experimental configurations. (**d**) Experimental linear wave damping rate per wavelength $\alpha$ for monochromatic wave propagation as a function of *f* for $h_g= 1.5\,\hbox {cm}$. $\alpha$ values for different waveguides (red symbols) with $a = 2.0$ ($*$), 2.5 (open diamond), 5.0 (open square) and 10 (open circle) cm are bounded between the computed $\alpha$ (blue symbols) for free one (open square) and two (open circle) dimensional wave propagation.

We measured the surface wave profile in space and time using an optical profilometry technique^30,31^ for different experimental parameters. From the set of surface wave profiles we reconstruct the wave envelope, to follow closely the fiber optical analogy proposed in the Introduction. These are constructed by averaging several images taken with the same time-lag between them. Note that the wavelength of the guided wave is larger than the one for the radiated wave, which is characterized by its propagation constant $\beta =2\pi /\lambda$. To characterize the waveguiding process, we fix $\delta \eta$ at 2 mm and $h_0$ at 2.5 cm. Thus, our experiments are set in the limit of steepness $\delta \eta /h_0$ and an Ursell number $\lambda ^{2}\delta \eta /(4\pi ^{2}h_0^{3})$ below 0.1, which allows the application of linear wave theory^18^. Hence, we can focus on the effect of experimental parameters $(f, h_g, a)$, systematically. First, we fix $a=2.5\,\hbox {cm}$ and $h_g=1.5\,\hbox {cm}$, and change *f * between 2.5 to $5.0\,\hbox {Hz}$, with a step of $0.5\,\hbox {Hz}$. We show some representative experimental results in Fig. 2. We observe that surface waves have a well defined bell-shaped profile in the *x*-axis, while propagating along the *y* direction. The amplitude of these trapped surface waves is, naturally, decreasing as they move away from the channel exit ($y=0$). As *f* increases, the distance the guided wave can propagate along the waveguide region decreases. We experimentally found that for $f= 5.0\,\hbox {Hz}$ almost no wave propagates along the waveguide region, observing an effective cut-off for propagating modes. This could be associated solely to viscous wave damping, which increases for higher frequencies^18^. We claim that the observed phenomena is mainly a consequence of waveguiding and not viscous dissipation. To prove this claim, we characterize experimentally the linear wave damping via its damping rate per wavelength $\alpha =\alpha (f,a,h_0)$, which is measured by fitting the wave amplitude’s spatial decay with an exponential function $\hat{\eta }_0\times \exp {(-\alpha y/\lambda )}$. This fitting scheme is performed along a line on the *y*-direction centered at the middle of the waveguide section. For $h_g$ = 1.5 cm, the measured $\alpha$ values remain confined between two bounds for all values of *a* and *f*, presented by blue symbols, as shown in Fig. 2d. The lower bound is set by the wave damping rate measured along a one-dimensional wave channel (6 cm wide), similar to the one described above where no two-dimensional diffraction can take place. The measured values for one dimensional propagation are within 10% of the ones computed from the theoretical one dimensional shallow water damping rate per wavelength^32^ $(2\pi /4h)\times (2\nu /\omega )^{1/2}$. The upper bound is set by the measured wave damping rate per wavelength of two-dimensional waves that propagate on an open tank after diffraction occurs (see Fig. 1b). In between these bounds, we observe the set of $\alpha$ values for different experimental waveguide configurations and frequencies. There is a large scatter of $\alpha$ values for $a<$ 5 cm and $f<$ 3 Hz, which can be attributed to modulations of the wave amplitude observed in Fig. 2a–c . Thus, $\alpha$ for guided waves are roughly 2 times smaller than the ones for a wave propagating over the open tank without waveguiding, which supports our claim of a one dimensional waveguiding dynamics.

**Figure 3:**
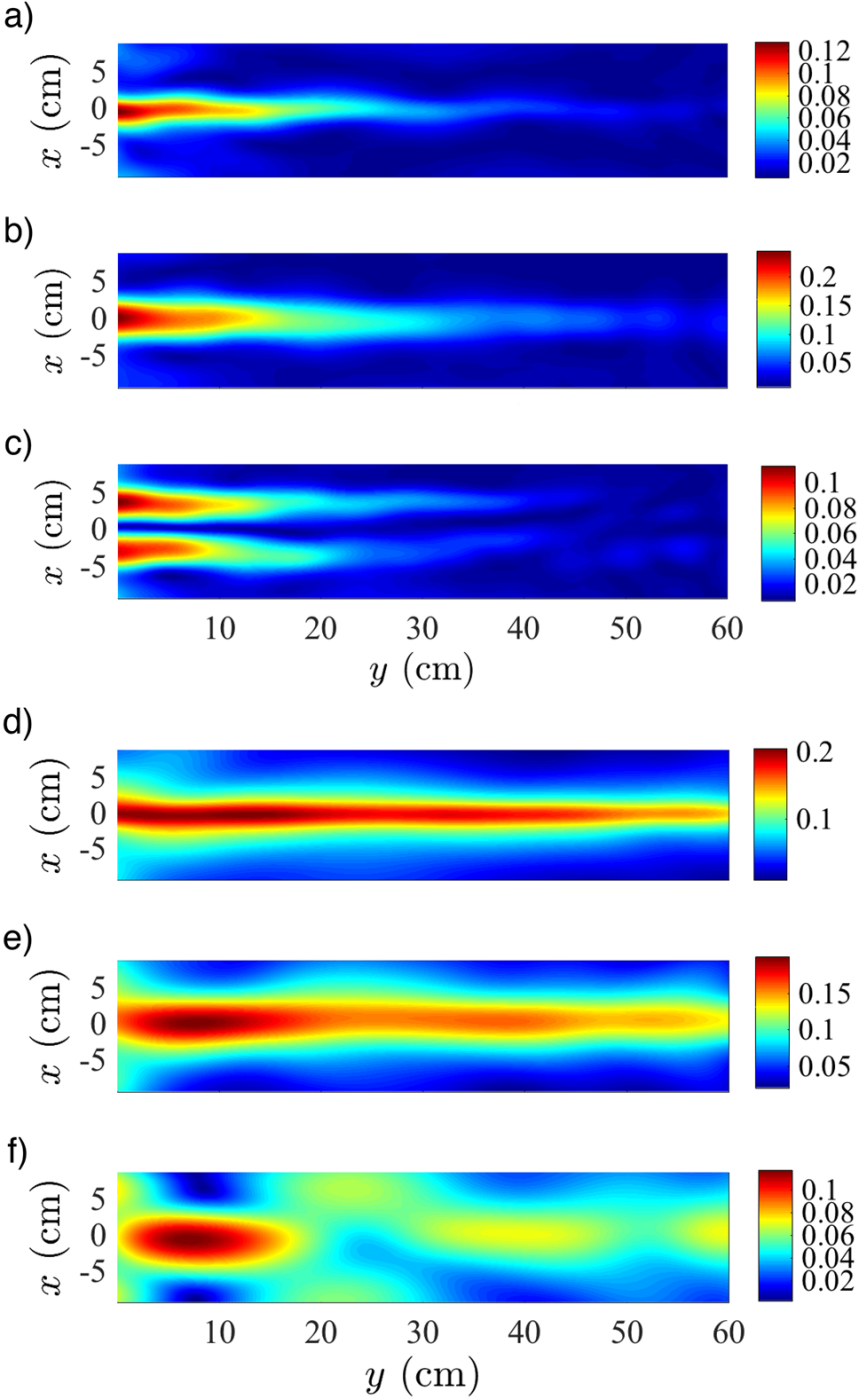
Wave envelope profiles for (**a**) $a = 2.5\,\hbox {cm}$, (**b**) $a = 5.0\,\hbox {cm}$ and (**c**) $a = 10.0\,\hbox {cm}$, with $\delta \eta = 2\,\hbox {mm}$, $h_g =1.5\,\hbox {cm}$, and $f = 4\,\hbox {Hz}$. Wave envelope profiles for (**d**) $h_g = 1.0\,\hbox {cm}$, (**e**) $h_g = 1.5\,\hbox {cm}$ and (**f**) $h_g = 2.2\,\hbox {cm}$, with $\delta \eta = 2\,\hbox {mm}$, $a=2.5\,\hbox {cm}$ and $f = 4\,\hbox {Hz}$.

Now, we fix $f= 4\,\hbox {Hz}$ and $h_g=1.5\,\hbox {cm}$, and run the experiment for three different bar widths (*a*), as shown in Fig. 3a–c. As *a* increases, we observe that the propagated wave along the waveguide region changes its transverse profile while it propagates along the *y*-axis: the effective propagation distance becomes smaller and transversal oscillations of the trapped wave profile appear, as it can be observed in Fig. 3a–c. These oscillations modify the profile, previously observed for narrower waveguides. In particular, the wider waveguide (see Fig. 3c) presents a profile associated to a first excited waveguide mode^3^, which also displays a different propagation constant. Finally, we set $f= 3\,\hbox {Hz}$ and $a=2.5\,\hbox {cm}$, and vary the bar height ($h_g$) to identify its effect on the guided wave amplitude. We explore three different values for $h_g$= 1.0, 1.5 and $2.2\,\hbox {cm}$, and present our results in Fig. 3d–f. For the smallest value of $h_g$ the wave is well guided and almost no transverse modulation is observed. Its propagation distance is comparable to the case where $h_g$ = 1.5 cm, if not larger. For $h_g$ = 2.2 cm, the wave is still guided although longitudinal oscillations are observed along the waveguide, as well as some wave radiation away from the waveguiding region.

## Theoretical modeling

To rationalize these observations, we focus on studying harmonic propagation, with a given angular frequency $\omega =2\pi f$, of surface water waves in the linear inviscid case. In the open wave tank, surface gravity wave motion is encoded in the velocity potential

1 $$\begin{aligned} \Phi (x,y,z,t)= \text {Re}\left[ \phi (x,y)\cosh {(k(z+h))} e^{-i\omega t}\right] , \end{aligned}$$

where *z* is the vertical coordinate normal to the bottom of the wave tank, as sketched in Fig. 1a. Here $\phi (x,y)$ is the solution of the Helmholtz equation

2 $$\begin{aligned} (\nabla ^{2}+k^{2})\phi (x,y)=0\ , \end{aligned}$$

where $\nabla$ is the bidimensional gradient and *k* is the wave vector chosen from the dispersion relation for surface gravity waves

3 $$\begin{aligned} \omega (k)=\sqrt{gk\tanh {(k h)}}\ . \end{aligned}$$

Deformations of the interface $\eta (x,y,t)$ from the flat surface at $z=0$ are then found by the relation

$$\begin{aligned} \eta (x,y,t)= \text {Re}\left[ -\frac{i\omega }{g}\phi (x,y) e^{-i\omega t}\right] \ . \end{aligned}$$

In our experiments, the local depth of the fluid changes from $h=h_0$ to $h=h_0-h_g$ over the waveguide, which changes the wave vector from $k=k_0$ to $k=k_g$. Thus, the ansatz (1) does not fulfill the boundary conditions where depth discontinuities develop, and the complete boundary value problem must be solved in order to find the observed waveguided modes. However, this issue can be sorted out if one deals with surface gravity waves in the shallow water limit. In this limit $k h\ll 1$, Eq. (3) becomes $k=\omega /\sqrt{gh}$ and the problem reduces to non-dispersive waves with different wave speeds inside ($\sqrt{g(h_0-h_g)}$) and outside ($\sqrt{gh_0}$) the waveguide region^18^. Assuming that the wave is guided in the *x*-direction while propagating along the *y*-axis, then $\phi (x,y)=\tilde{\phi }(x)e^{i\beta y}$, and thus Eq. (2) turns to

4 $$\begin{aligned} \left[ \partial _{x}^{2}+(k_0^{2}-\beta ^{2})\right] \tilde{\phi }(x)=0\ , \end{aligned}$$

outside the waveguide region, and

5 $$\begin{aligned} \left[ \partial _{x}^{2}+(k_g^{2}-\beta ^{2})\right] \tilde{\phi }(x)=0\ , \end{aligned}$$

inside. Here $\tilde{\phi }(x)$ represents the transversal profile of the guided mode and $\beta$ corresponds to its propagation constant. $\tilde{\phi }(x)$ satisfies Eqs. (4) and (5) with $\tilde{\phi }$ and $\partial _x\tilde{\phi }$ continuous at the edges of the waveguide region. From these equations, we find symmetric modal solutions for $\tilde{\phi }(x)$ when

6 $$\begin{aligned} \sqrt{\frac{\beta _s^{2}-k_0^{2}}{k_g^{2}-\beta _s^{2}}}=\tan {\left[ \sqrt{\left( k_g^{2}-\beta _s^{2}\right) }\ \frac{a}{2}\right] } \end{aligned}$$

is satisfied for a propagation constant $\beta =\beta _s$, where “s” stands for “symmetric”. For asymmetric modes, this relation is similar but changing “$\tan$” for “$-\cot$” and $\beta _s$ for $\beta _a$. These relations allow us to find the possible mode propagation constants $\beta =\{ \beta _s,\beta _a\}$ in the wave vector band $k_0<\beta <k_g$. The relation above is completely analog to what is found in optical waveguides^4,5^ where waveguiding occurs in regions with a larger refractive index as the light velocity becomes smaller. Optical guided modes have a $\cos$-like profile inside the waveguide region and an exponentially decaying wave function (evanescent field) outside of it. Therefore, our water waveguide is completely equivalent to an optical waveguide, specifically to the case having a one dimensional step-like refractive index profile, a concept used in different physical contexts^2,37^.

**Figure 4:**
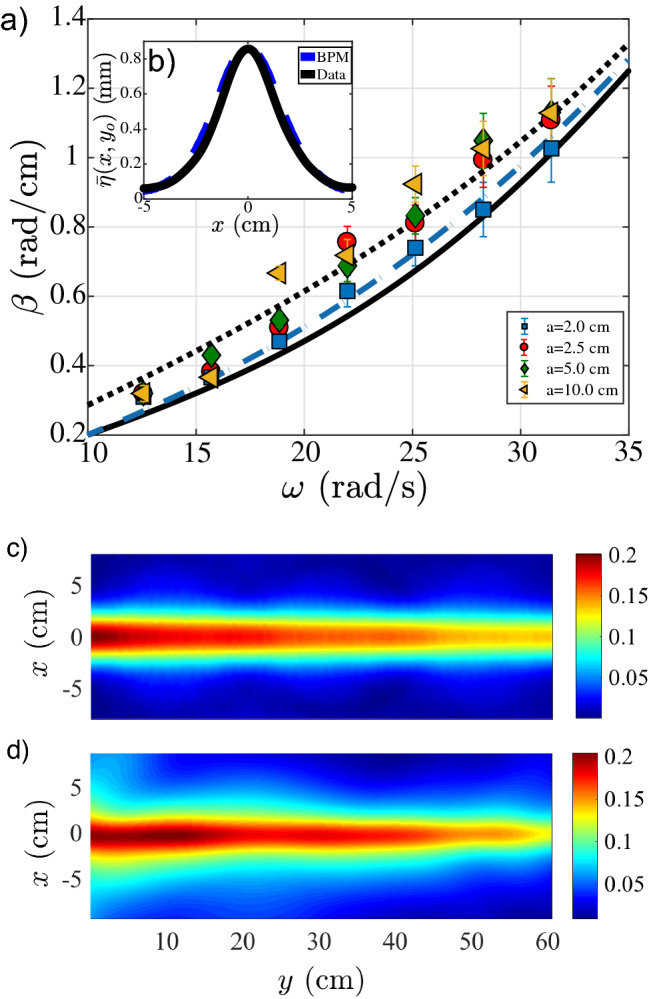
(**a**) Experimental values for $\beta$ as a function of $\omega$ for $a= 2.0$ (open square), 2.5 (open circle), 5.0 (open diamond) and 10.0 (open left angled triangle) cm. Lines show the theoretical dispersion relation curves for gravity surface waves $\omega =\omega (k)$ with $k=k_0$ when $h=h_0$ (continuous line) and $k=k_g$ when $h=h_0-h_g$ (dotted line). These curves set the boundaries of the wave band $k_0<\beta <k_g$. In between these boundaries, a dashed line shows numerically computed $\beta _s$ as a function of $\omega$, obtained from Eq. (6) for $a= 2\,\hbox {cm}$. (**b**) Numerical (continuous line) and experimental (dashed line) profiles for the first propagative mode, for $a=2.5\,\hbox {cm}$, $h_g=1\,\hbox {cm}$, $f= 3\,\hbox {Hz}$, and $\delta \eta = 2\,\hbox {mm}$. (**c**) Numerical and (**d**) experimental wave envelopes for $a=2.5\,\hbox {cm}$, $h_g=1\,\hbox {cm}$, $f= 3\,\hbox {Hz}$, and $\delta \eta = 2\,\hbox {mm}$.

When shallow water theory can not be used, one needs to solve the above problem including the condition of zero normal derivative along the entire bottom surface for $\Phi$^33–35^. In this case, a more complex relation between the waveguide parameters is found. We have computed $\beta$ using shallow water theory and the complete shelf model from Miles^35^, applied to our problem (see “Methods” section). In the case of narrow waveguides ($a=2.0\,\hbox {cm}$), only symmetric modes are excited. For wider waveguides ($a=10.0\,\hbox {cm}$) and $f > 3.0\,\hbox {Hz}$ ($\omega > 6\pi \,\hbox {rad/s}$), asymmetric modes can be excited as well. This information is compiled in Tables 1 and 2, where for narrow waveguides we observe only symmetric states, while for wider ones we observe the appearance of asymmetric states above $f > 3.0\,\hbox {Hz}$. In Fig. 4a we show the experimental $\beta$ values as a function of $\omega$, for $h_g=1.5\,\hbox {cm}$ and for 4 different waveguide widths. We also show the dispersion relation (3) inside (continuous line) and outside (dotted line) the waveguide region, which sets the wave vector band $k_0<\beta <k_g$. We observe an excellent agreement between the experimentally measured $\beta$ values (symbols) and the numerical calculation of Eq. (6), for narrow waveguides (see dashed line in Fig. 4a). This case is simpler due to the absence of higher-order modes in the measured frequency range, as shown in Table 1. For wider waveguides, we observe a mismatch between the numerically computed $\beta$ values (shown in Table 2) and the experimental ones for $\omega > 21.99\,\hbox {rad/s}$ [$f = 3.5\,\hbox {Hz}$]. This can be the result of the development of asymmetric as well as symmetric modes, propagating along the waveguide region. In addition, Fig. 4b shows a comparison between the symmetric mode profiles obtained numerically and experimentally for a narrow waveguide ($a= 2.5\,\hbox {cm}$).

**Table 1:** Computed propagation constants for symmetric ($\beta _s$) modes, for $\{a,h_g\}=\{2.0,1.5\}\,\hbox {cm}$.

$\omega$ (rad/s)	12.57	15.71	18.85	21.99	25.13	28.27	31.42
$\beta _s$ (rad/cm)	0.27	0.36	0.46	0.56	0.69	0.85	1.02

**Table 2:** Computed propagation constants for symmetric ($\beta _s$) and asymmetric ($\beta _a$) modes, for $\{a,h_g\}=\{10.0,1.5\}\,\hbox {cm}$.

$\omega$ (rad/s)	12.57	15.71	18.85	21.99	25.13	28.27	31.42
$\beta _s$ (rad/cm)	0.30	0.40	0.50	0.62	0.75	0.90	1.06
$\beta _a$ (rad/cm)	–	–	–	0.56	0.69	0.84	1.02

Following the equivalence between shallow water and optics, we compute the evolution of the guided water wave by implementing a beam propagation method (BPM) which solves numerically Eq. (2). This method, widely used in optics, allows us to track the envelope evolution along the waveguide. We include dissipation effects by multiplying directly the numerically obtained wave field on each spatial position with a damping exponential factor $\exp {(-\alpha y/\lambda )}$. In Fig. 4c we show a numerical example, including an exponentially decaying factor due to dissipation, and compare it to its experimental counterpart in Fig.  4d. The numerical result is completely symmetric in the *y*-direction, due to the absence of inhomogeneities while the experimental image is slightly asymmetric due to different background waves propagating through the open tank. Both figures display an excellent agreement, which corroborates water waveguiding in an open fluid layer.

Finally, following Eq. (3) and considering our experimental configuration for $\omega > 31.42\,\hbox {rad/s}$ ($f = 5\,\hbox {Hz}$), we get $k_0=k_g$. Therefore, as we enter the deep water limit outside and inside the waveguide, waveguiding becomes forbidden. The absence of an effective velocity contrast does not allow the excitation of guided modes. This is in agreement with the experimentally obtained data presented in Fig. 4a, where the existence region shrinks for $f> 5\,\hbox {Hz}$.

## Conclusions

In conclusion, we have shown experimentally, theoretically and numerically water waveguiding, without channeling, which can be envisioned as an optical analogue. Thus, all the machinery built for optics can be applied directly on surface water wave problems, considering slight modifications such as the inclusion of depth effects^36^, and viscous damping. From this point, water waveguiding on curved waveguides or solitonic propagation can be pushed towards a complete control of guided surface wave packets. Additionally, evanescent wave fields can be used to generate a coupling mechanism to transfer surface wave energy in the transversal direction, as discrete optics research has shown^37^. Harnessing trapped surface wave energy could be of great industrial and environmental utility, by presenting solutions for controlling water wave phenomena in different scales (including tsunamis and heavy seas), which is nowadays relevant due to the evident change in global weather^38^. This also shows an important avenue for future basic and applied research using water waveguiding in different configurations, where our present work could be just the beginning of a revival for such a wonderful new area of development in physics.

## Methods

### Profilometry

To access the spatio-temporal wave profile an optical technique adapted for free surface characterization is implemented^30,31^. Different image sequences are acquired for a given *f* and $\delta \eta$, centered around the waveguide. Each image spanned $15.5 \times 62.5\,\hbox {cm}^{2}$ with a 0.04 pix/cm sensitivity. The vertical resolution is the projected pixel size. The time step between images is set by the camera’s acquisition frequency at 30 fps, and the number of images per sequence is set at 1800. Our accessible wave numbers are limited by the wavelength of the projected light fringes used in the method, which is set at 0.5 cm. Figure 1a shows an sketch of the fringes generated by a projector, including the camera which collects light from the water surface. The average envelope of the guided surface wave is reconstructed by Fourier filtering each image pixel in time and then computing its *rms* fluctuations, which is averaged for a set of images. Figure 1b,c display stroboscopic wave envelopes of the experimental surface wave pattern without and with a waveguiding bottom, respectively. Note that the wavelength of the guided wave, characterized by its propagation constant $\beta =2\pi /\lambda$, is larger than the one for the radiated wave.

### Deep water calculations for waveguiding

When the shallow water limit is not fullfilled, a theoretical derivation for intermediate and deep water wave calculation must be performed. In what follows, we compute the possible guided modes and propagation constants which are presented in Table 1. The problem is based on the calculation of $\Phi (x,y,z,t)$ which satisfies $\nabla ^2 \Phi = 0$, with boundary conditions, that read

7 $$\begin{aligned} \frac{\omega ^{2}}{g}\Phi +\partial _z\Phi= & {} 0,\ \ \ \ z=0; \end{aligned}$$

8 $$\begin{aligned} \partial _z\Phi= & {} 0, \ \ \ \ z=-h_0, |x|>|a/2|; \end{aligned}$$

9 $$\begin{aligned} \partial _z\Phi= & {} 0, \ \ \ \ z=-h0-h_g, |x|\le a/2; \end{aligned}$$

10 $$\begin{aligned} \partial _x\Phi= & {} 0, \ \ \ \ x=\pm a/2, -h_0<z<-h0-h_g; \end{aligned}$$

which are valid in the inviscid linear regime^35^. Assuming that $\Phi$ can be decomposed (similarly as in the main text) as $\Phi =\text {Re}(\tilde{\phi }(x)\theta (z)\exp {(i\beta y)})$, it is found that

11 $$\begin{aligned} \dfrac{ \tilde{\phi }''(x)}{\tilde{\phi }(x)}+ \dfrac{\theta ''(z)}{\theta (z)}-\beta ^2 = 0, \end{aligned}$$

where $(\cdot )'$ stands for derivatives. Due to this separation of variables, using $\partial _z \Phi =0$ on $z=-h_i$, with $h_i$ is either $h_0$ or $h_0-h_g$, one finds that

12 $$\begin{aligned} \theta (z) = \psi (z,\kappa ) = 2^{\frac{1}{2}}\left[ h-\frac{g}{\omega ^2}\sin ^2(\kappa h)\right] ^{-\frac{1}{2}}\cos [\kappa (h-z)]\ , \end{aligned}$$

with the dispersion relation

13 $$\begin{aligned} g\kappa \tan (\kappa h)+\omega ^2=0,\ \end{aligned}$$

where $\psi (z,\kappa )$ are the functions used by Miles^35^, which are orthonormal within $[-h_i,0]$. There are a numerable set of $\kappa \in \mathbb {R}$ solutions of Eq. (13), and only one pure imaginary one. This means that Eq. (11) reads

14 $$\begin{aligned} \dfrac{\tilde{\phi }''(x)}{\tilde{\phi }(x)}= \beta ^2+\kappa ^2. \end{aligned}$$

Each $\kappa$ defines a $\tilde{\phi }$ and the sign of $(\beta ^2+\kappa ^2)$ determines its oscillatory or decaying nature. Thus, in 2 different regions (region 1 where $h=h_0$ and region 2 where $h=(h_0-h_g)$) there will be real (*k* and $\tilde{k}$, respectively) and imaginary ($i k_1$ and $ik_2$, respectively) solutions. It must be noticed that in order to construct a guided solution one needs that $(\beta ^2 -k_1^2)>0$ outside the waveguiding region and $(\beta ^2-k_2^2)<0$ inside the waveguiding region.

Applying that away from the waveguiding region $\tilde{\phi }(x \rightarrow \pm \infty ) = 0$, then the symmetric solution for $\Phi$ in the *x*–*z* plane must read

15 $$\begin{aligned} \displaystyle \tilde{\phi }(x)\theta (z) = \left\{ \begin{array}{lcc} \displaystyle \sum _{k} C_1(k) e^{-\sqrt{k^2+\beta ^2}|x|}\psi _1(z,k) + E_1 e^{-\sqrt{\beta ^2-k_1^2}|x|} \psi _1(z,ik_1)&{} \ \ \ \ &{} |x| \ge a/2 \\ \\ A_2\cos \left( \sqrt{k_2^2-\beta ^2}|x|\right) \psi _2(z,ik_2) + \displaystyle \sum _{\tilde{k}}C_2(\tilde{k})\cosh \left( \sqrt{\tilde{k}^2+\beta ^2}|x|\right) \psi _2(z,\tilde{k}) &{} \ \ \ \ &{} |x|<a/2 \end{array} \right. \end{aligned}$$

which can be used to compute the spatial derivatives of $\Phi$. As both $\Phi$ and $\partial _x \Phi$ must be continuous at $x=\pm a/2$, we can relate $C_2(\tilde{k})$ and $A_2$ to $C_1({k})$ and $E_1$. Using the fact that $\psi _2(z,\tilde{k})$ and $\psi _2(z,ik_2)$ in $[0,h_0-h_g]$ are orthonormal by construction, one obtains 4 different equations via projecting onto Miles’s functions which are used to equate the expressions for $C_2(\tilde{k})$ and $A_2$, allowing us to compute $\beta$

16 $$\begin{aligned}&A \left[ \dfrac{1}{\cosh ((a/2)\sqrt{\tilde{k}^2+\beta ^2})}+\dfrac{\sqrt{k^2+\beta ^2}}{\sqrt{\tilde{k}^2+\beta ^2}\sinh ((a/2)\sqrt{\tilde{k}^2+\beta ^2})}\right] \nonumber \\&\qquad \times \left[ \dfrac{1}{\cos ((a/2)\sqrt{k_2^2-\beta ^2})}-\dfrac{\sqrt{\beta ^2-k_1^2}}{\sqrt{k_2^2-\beta ^2}\sin ((a/2)\sqrt{k_2^2-\beta ^2})} \right] \nonumber \\&\quad =B\left[ \dfrac{1}{\cosh ((a/2)\sqrt{\tilde{k}^2+\beta ^2})}+\dfrac{\sqrt{\beta ^2-k_1^2}}{\sqrt{\tilde{k}^2+\beta ^2}\sinh ((a/2)\sqrt{{\tilde{k}^2+\beta ^2}})} \right] \nonumber \\&\qquad \times \left[ \dfrac{1}{\cos ((a/2)\sqrt{k_2^2-\beta ^2})}-\dfrac{\sqrt{k^2+\beta ^2}}{\sqrt{k_2^2-\beta ^2}\sin ((a/2)\sqrt{k_2^2-\beta ^2})}\right] \end{aligned}$$

due to $C_1(k)>0$. Here we have used that $A = (k^2-\tilde{k}^2)^{-1}(k_1^2-k_2^2)^{-1}$ and $B = (k^2+k_2^2)^{-1}(k_1^2+\tilde{k}^2)^{-1}$. In the above calculations we have solved the symmetric case, and thus $\beta =\beta _s$. A similar calculation can be done for the asymmetric case $\beta =\beta _a$ by solving

17 $$\begin{aligned}&A \left[ \dfrac{1}{\sinh ((a/2)\sqrt{\tilde{k}^2+\beta ^2})}+\dfrac{\sqrt{k^2+\beta ^2}}{\sqrt{\tilde{k}^2+\beta ^2}\cosh ((a/2)\sqrt{\tilde{k}^2+\beta ^2})}\right] \nonumber \\&\qquad \times \left[ \dfrac{1}{\sin ((a/2)\sqrt{k_2^2-\beta ^2})}+\dfrac{\sqrt{\beta ^2-k_1^2}}{\sqrt{k_2^2-\beta ^2}\cos ((a/2)\sqrt{k_2^2-\beta ^2})} \right] \nonumber \\&\quad =-B\left[ \dfrac{1}{\sinh ((a/2)\sqrt{\tilde{k}^2+\beta ^2})}+\dfrac{\sqrt{\beta ^2-k_1^2}}{\sqrt{\tilde{k}^2+\beta ^2}\cosh ((a/2)\sqrt{\tilde{k}^2+\beta ^2})} \right] \nonumber \\&\qquad \times \left[ \dfrac{1}{\sin ((a/2)\sqrt{k_2^2-\beta ^2})}+\dfrac{\sqrt{k^2+\beta ^2}}{\sqrt{k_2^2-\beta ^2}\cos ((a/2)\sqrt{k_2^2-\beta ^2})}\right] . \end{aligned}$$

By solving Eqs. (16) and (17), different values of the propagation constant $\beta$ can be found for both the symmetric and asymmetric cases, which are the values we used to construct the tables displayed in the main text. Moreover, once the value of $\beta$ is known, a spatial profile corresponding to that mode can be calculated.

## Data Availability

The datasets generated during and/or analyzed during the current study are available from the corresponding author on reasonable request.
